# Pixel-Level Recognition of Trace Mycotoxins in Red Ginseng Based on Hyperspectral Imaging Combined with 1DCNN-Residual-BiLSTM-Attention Model

**DOI:** 10.3390/s24113457

**Published:** 2024-05-27

**Authors:** Biao Liu, Hongxu Zhang, Jieqiang Zhu, Yuan Chen, Yixia Pan, Xingchu Gong, Jizhong Yan, Hui Zhang

**Affiliations:** 1 College of Pharmaceutical Science, Zhejiang University of Technology, No. 18, Chaowang Road, Hangzhou 310014, China; liubiao812@zjut.edu.cn (B.L.); 2112007127@zjut.edu.cn (H.Z.); zhujieqiang@zjut.edu.cn (J.Z.); 2112107116@zjut.edu.cn (Y.C.); 13505822782@163.com (Y.P.); 2 Pharmaceutical Informatics Institute, College of Pharmaceutical Sciences, Zhejiang University, Hangzhou 310058, China; gongxingchu@zju.edu.cn

**Keywords:** hyperspectral imaging, red ginseng, mycotoxins, pixel-level recognition, deep learning, 1DCNN-ResBiLSTM-Attention

## Abstract

Red ginseng is widely used in food and pharmaceuticals due to its significant nutritional value. However, during the processing and storage of red ginseng, it is susceptible to grow mold and produce mycotoxins, generating security issues. This study proposes a novel approach using hyperspectral imaging technology and a 1D-convolutional neural network-residual-bidirectional-long short-term memory attention mechanism (1DCNN-ResBiLSTM-Attention) for pixel-level mycotoxin recognition in red ginseng. The “Red Ginseng-Mycotoxin” (R-M) dataset is established, and optimal parameters for 1D-CNN, residual bidirectional long short-term memory (ResBiLSTM), and 1DCNN-ResBiLSTM-Attention models are determined. The models achieved testing accuracies of 98.75%, 99.03%, and 99.17%, respectively. To simulate real detection scenarios with potential interfering impurities during the sampling process, a “Red Ginseng-Mycotoxin-Interfering Impurities” (R-M-I) dataset was created. The testing accuracy of the 1DCNN-ResBiLSTM-Attention model reached 96.39%, and it successfully predicted pixel-wise classification for other unknown samples. This study introduces a novel method for real-time mycotoxin monitoring in traditional Chinese medicine, with important implications for the on-site quality control of herbal materials.

## 1. Introduction

The safety issues of traditional Chinese medicine (TCM) have been attracting significant attention, especially in regard to the detection of mycotoxins, which has emerged as a focal point of concern. Red ginseng is the dried root and rhizome of the cultivated plant *Panax ginseng* C.A. Mey, which belongs to the Araliaceae family. It is a type of processed ginseng product with significant and unique physiological activities. The process enhances its medicinal properties, provides various benefits such as anti-fatigue characteristics, blood pressure regulation, and immune system enhancement [1]. Red ginseng has been used as a medicinal plant for thousands of years in countries such as China, South Korea, and Japan [2]. However, improper handling during the cultivation, harvest, storage, and processing of ginseng, as well as the preparation and production of red ginseng, can lead to fungal contamination and the production of mycotoxins [3,4,5]. Mycotoxins are toxic secondary metabolites, and currently, several mycotoxins have been detected in moldy ginseng and its processed products, including aflatoxins (AFs) [6,7], ochratoxin A (OTA) [8,9], zearalenone (ZEN) [10], and fumonisins [11]. These mycotoxins possess acute toxicity, including mutagenic, carcinogenic, and teratogenic effects [12]. Consequently, the presence of mycotoxins in moldy ginseng and its processed products has become a critical safety concern, drawing significant attention from the public [13].

Currently, the commonly used methods for detecting mycotoxins in TCM include high-performance liquid chromatography (HPLC) [14], enzyme-linked immunosorbent assays (ELISAs) [15], and liquid chromatography-mass spectrometry (LC-MS) [16]. These methods offer accurate quantification and good reproducibility. However, they are associated with complex sample preparation, high detection costs, slow detection speed, and chemical methods that result in sample destruction, rendering them unsuitable for re-entry into production. Therefore, there is an urgent need for a rapid, accurate, and real-time online detection method for mycotoxins in TCM, which holds significant importance for the quality control of herbal medicines and human health.

Hyperspectral imaging (HSI) technology integrates spectral and machine vision techniques, allowing the simultaneous analysis of both spectral and image information related to the quality attributes of the target object. It is a non-polluting, fast, and non-destructive detection technique [17,18,19]. HSI consists of hundreds of spectral bands, and due to the distinct molecular structures, they can clearly reflect the variations of the absorption peaks of compounds in different bands. By extracting these features, it is possible to qualitatively determine the presence of mycotoxins. Regarding the detection of mycotoxins using HSI, there have been some reports in the agricultural field. Zhang et al. [20] utilized machine vision to detect aflatoxin B_1_ (AFB_1_) in corn and compared the modeling performance of four pre-processing methods, four feature wavelength extraction methods, and three chemometrics methods. They achieved a detection accuracy of 87.3% by using Savitzky-Golay (SG) smoothing for pre-processing and combining the successive projections algorithm (SPA) for feature wavelength extraction with a support vector machine (SVM) model. Renata et al. [21] employed short-wave infrared (SWIR) HSI technology to detect two species of Fusarium fungi in corn kernels. They developed a principal component analysis (PCA) combined with a partial least squares-discriminant analysis (PLS-DA) model to classify contaminated corn kernels, achieving a classification accuracy of 92.41%. The aforementioned studies were based on traditional machine learning models for mycotoxin detection. However, for HSI with large amounts of data, traditional machine learning algorithms usually require data feature selection and decomposition. In contrast, deep learning demonstrates more robust capabilities for large-scale data processing and feature extraction.

In recent years, with the development of deep-learning algorithms, they have been widely applied in the field of HSI [22,23,24,25,26]. Deep learning has also been used for the detection of aflatoxin in HSI images in agriculture. Gao et al. [27] employed VGG-16 to classify each pixel on peanut and corn crops, distinguishing whether AFB_1_ was present. The detection accuracies for peanut, corn, and the mixed dataset were 96.35%, 92.11%, and 94.64%, respectively. Han et al. [28] designed a one-dimensional convolutional neural network (1D-CNN) for detecting AFB_1_ in peanuts. By reshaping the spectral images and combining deep-learning feature extraction, the overall recognition rate exceeded 95%. Liu et al. [29] established DeepLab v3, SegNet, Unet, and Hypernet deep-learning models for identifying moldy peanuts. By utilizing feature enhancement techniques, the average recognition accuracy of moldy peanuts was raised to 92.07%. Currently, deeply integrated models, such as convolutional neural networks (CNNs), long short-term memory (LSTM), CLSTM (a hybrid model of CNN and LSTM), or auxiliary attention machine (AM) models like ACNN (AM and CNN), ALSTM (AM and LSTM), and ACLSTM (a deep-learning model combining AM, CNN, and LSTM), have been successfully applied to the origin classification and content determination of TCM [24,25]. However, there have been no reports on the identification of fungal toxins in TCM.

Currently, in the process of using hyperspectral detection for mycotoxin recognition, a common approach to simulate the presence of mycotoxins in the target is to artificially add a single mycotoxin standard substance (e.g., AFB_1_) for modeling. However, in real-life situations, food or TCM may be contaminated with mixtures of mycotoxins, such as AFs and OTA, when they become moldy. Therefore, solely detecting single artificially added standard substances may deviate significantly from the actual scenario. Furthermore, in the currently reported studies, the recognition targets are limited to the target object and mycotoxins without considering the presence of interfering impurities that may affect the judgment. Simple binary classification models focusing only on the target object and mycotoxins are prone to misclassify interfering impurities as one of the two categories, leading to potential false positives. At present, in the field of TCM, the application of HSI to detect mycotoxins in herbal materials is still unexplored, leaving this area relatively unknown.

In this study, red ginseng was taken as an example; the pixels containing mycotoxins in red ginseng were extracted and used for training in a deep-learning model. Additionally, to further consider the interference from other substances present in real-world scenarios during the process and storage of red ginseng, this study introduced interfering impurities that may be encountered in the process. The corresponding pixel spectra were also extracted and used for training. Finally, red ginseng samples with different mildew levels and added interference impurities were identified at the pixel level and the results were visualized for analysis.

## 2. Materials and Methods

### 2.1. Preparation of the Red Ginseng Samples

In real-life situations, the mycotoxins produced in moldy red ginseng are a mixture of various mycotoxins rather than a single pure substance. Merely relying on manually adding mycotoxin standard substances cannot fully represent the actual scenario of red ginseng molding. Therefore, in this experiment, an environment conducive to red ginseng spoilage was established to simulate the process of mycotoxin production and obtain realistically moldy red ginseng samples.

The red ginseng used in the experiment was provided by Chiatai Qingchunbao Pharmaceutical Co., Ltd. (HangZhou, China). One hundred and fifteen samples of red ginseng with good appearance and uniform quality were randomly divided into five groups. A constant temperature of 30 °C and 85% humidity were set to simulate a favorable growth environment for mycotoxin production [30]. Each of the five groups, including both artificially moldy red ginseng and naturally moldy red ginseng under real conditions, was cultured in the constant temperature and humidity chamber for 0, 2, 4, 6, and 8 days, respectively, to obtain the samples of red ginseng with mycotoxins.

### 2.2. Hyperspectral Imaging System and Data Acquisition

The configuration of the HSI instrument is shown in Table S1. Before using the HSI instrument, pre-heating was required. Additionally, the camera needed to be focused, and the speed of the moving platform was finely adjusted to ensure the clear and complete image acquisition of the samples. The specific parameters for the red ginseng sample using the HSI instrument are as follows: the camera exposure time was set to 16 ms, and the platform moving speed was 2.6 mm·s^−1^. This resulted in a hyperspectral data cube with a length of 1000 pixels and a width of 640 pixels. Moreover, 512 bands were selected within the range of 898 nm to 1751 nm with an interval of 1.67 nm. Subsequently, the blank channels of the HSI camera were removed, resulting in a final short-wave infrared spectrum range of 900 to 1749 nm and 510 bands. A total of one hundred and fifteen HSI images of the red ginseng samples were acquired.

The obtained reflectance images were affected by different factors: the illumination source, the dark current of the charge-coupled device (CCD) camera, and the different physical configurations of the hyperspectral imaging system. Therefore, a whiteboard reference image was acquired using a standard diffuse reflectance whiteboard with a specific reflectance, while the blackboard reference image was obtained with the light source completely turned off and the camera lens covered. The acquired hyperspectral images were calibrated with the black and white plate reference images as follows [19]:

(1) $$R_{C}=\left(R_{0}-R_{dark}\right)/\left(R_{white}-R_{dark}\right)$$

where *R_c_* is the calibrated reflectance image, *R_0_* is the raw reflectance image, *R_white_* is the white reference image, and *R_dark_* is the dark reference image.

### 2.3. ROI Selection and Pixel Extraction

The selection of the region of interest (ROI) was typically accomplished using the threshold segmentation method. In this method, the image was converted to a grayscale image, and a pre-defined threshold (in this study, the highest reflectance was in the 130th band, and the lowest reflectance was in the 340th band) was used to differentiate the target objects from the background and noise in the image [31]. However, when the grayscale values of the target pixels are close to those of the background pixels, the threshold segmentation method may lead to erroneous labeling. As the boundary pixels of the mycotoxins and red ginseng in this study were relatively close, Labelme software (v.5.4.1) was used for ROI selection. Labelme is an image annotation tool used to mark target objects and their coordinates. With this software, the regions of red ginseng were labeled as “Red ginseng”, while the regions of mycotoxins were labeled as “Mycotoxin”. The corresponding positions and label information were saved in a “json” file for further processing.

Using the coordinate information of the target regions from the aforementioned “json” file, the ROI of red ginseng and the mycotoxin in the hyperspectral images of red ginseng were determined. Subsequently, Python was used to extract the pixels from different ROIs. The specific process of pixel extraction can be found in the Supplementary Materials.

After pixel extraction, 400 pixels were randomly selected from the red ginseng region and mycotoxin region to establish the red ginseng mycotoxin (R-M) dataset. The Kennard-Stone algorithm was then applied to divide the pixels into the training set and the test set in a 2:1 ratio.

### 2.4. Training Model Selection

#### 2.4.1. Deep-Learning Model for CNNs

The 1D-CNN model was composed of four convolutional layers, each with a distinct number of convolutional kernels: 256, 128, 64, and 32, respectively. The kernel size was uniformly set to 3, and “same” padding was employed. The activation function utilized throughout the model was the Swish function. After each convolutional layer, a series of additional layers were applied for enhanced model performance and to address the risk of overfitting. Specifically, the sequence following each convolutional layer included a batch normalization layer, a MaxPooling1D layer, and a dropout layer. These additional layers contributed to the overall regularization of the model, ensuring better generalization and mitigating overfitting concerns. The operation of the Conv1D layer followed by the Swish activation function was performed as follows:

(2) $$y_{j}^{\left(k\right)}={Swish}_{1,2,3\in h}\left[\sum_{i=1}^{N_{\left(k-1\right)}}Conv1D\left(x_{i}^{\left(k-1\right)},W_{i,j}^{\left(k\right)}\right)+b_{j}^{\left(k\right)}\right]$$

(3) $$Swish\left(x\right)=x*sigmoid(x)$$

where $y_{j}^{\left(k\right)}$ denotes the output at position j in the *k*-th layer, the Swish activation function applied to the sum of convolutional operations. The sum $\sum_{i=1}^{N_{\left(k-1\right)}}$ aggregates the results of the convolutional operations over the input features from the previous layer (*k* − 1). $Conv1D\left(x_{i}^{\left(k-1\right)},W_{i,j}^{\left(k\right)}\right)$ represents the convolution operation, where $x_{i}^{\left(k-1\right)}$ is the *i*-th input feature, and $W_{i,j}^{\left(k\right)}$ is the corresponding convolutional kernel. Lastly, $b_{j}^{\left(k\right)}$ is the bias term associated with the *j*-th output channel.

#### 2.4.2. Deep-Learning Model for ResBiLSTM

The architectural composition of the ResBiLSTM model was designed to enhance its capacity to capture intricate temporal dependencies within sequential data, a crucial aspect for effective feature extraction in deep learning [32]. Leveraging bidirectional long short-term memory (BiLSTM) layers with 64 hidden units each, the model exploited both forward and backward temporal information to discern the spectra. A distinctive feature of the ResBiLSTM model lies in the incorporation of residual connections, wherein the output of the bidirectional LSTM layers is fused with the original input sequence. This strategic integration mitigates the vanishing gradient problem, facilitating the more robust learning of input representations. The mathematical equation for performing this task through the Bi-LSTM unit is explained as follows:

(4) $$\vec{h_{n}}=\sigma \left(W_{1}x_{n}+W_{2}\vec{h_{n-1}}\right)\times tanh(C_{n})$$

(5) $$\vec{h_{n}}=\sigma \left(W_{3}x_{n}+W_{4}\overset{\leftarrow }{h_{n-1}}\right)\times tanh({C\prime }_{n})$$

(6) $$h_{n}^{\left[p\right]}=W_{5}\vec{h_{n}}+W_{6}\overset{\leftarrow }{h_{n}}$$

where $\vec{h_{n}}$ represents the forward hidden state for number n, $\overset{\leftarrow }{h_{n-1}}$ represents the backward hidden state for number *n* − 1, $h_{n}^{\left[p\right]}$ represents the final hidden state for number *n*, σ is the sigmoid activation function, $W_{1}$, $W_{2}$, $W_{3}$, $W_{4}$, and $W_{5}$ are weight matrices, and $C_{n}$ is the cell state for number *n*.

#### 2.4.3. Deep-Learning Model for the Attention Mechanism 

The attention layer, a vital component of a deep-learning model, plays a pivotal role in capturing intricate dependencies within sequential data [33]. Initialized using the Xavier uniform function for optimal weight initialization, the layer’s adaptability was enriched by configurable options such as weight regularization, bias regularization, weight constraints, and bias constraints, offering efficient regularization and adaptability. During the construction phase, a three-dimensional weight tensor (W) was dynamically generated to conform to the input shape, ensuring seamless alignment with varying data dimensions. The layer supported masking for handling sequences of variable lengths. Attention scores were computed through matrix operations, followed by hyperbolic tangent activation and exponentiation. The softmax function was applied for normalization, resulting in attention weights that modulated the input sequence. The thoughtful design and configurable parameters of the attention layer contributed significantly to capturing and highlighting crucial information in the sequential data, enhancing the model’s expressive power and performance as follows:

(7) $$e_{t}=f\left(y_{t}\right),\;\alpha_{t}=\frac{exp(e_{t})}{\sum_{i=1}^{T}exp(e_{i})},\;C=\sum_{t=1}^{T}\alpha_{t}y_{t}$$

where the function f expression can be learned via backpropagation through the fully connected layer, and the weight $\alpha_{t}$ constantly changes during the training process, and as the training progresses, the feature information *C* gradually becomes more representational.

#### 2.4.4. 1DCNN-Residual-BiLSTM-Attention Model

**Figure 1:**
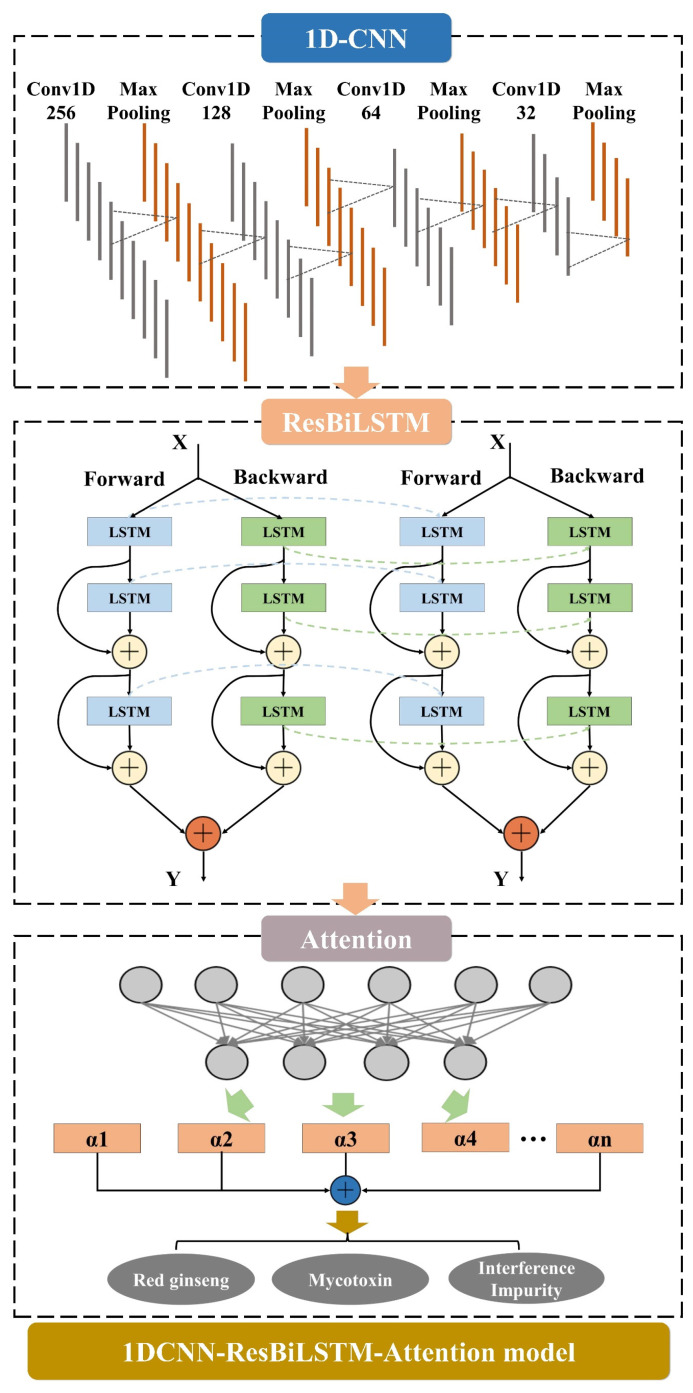
1DCNN-ResBiLSTM-Attention model. The 1DCNN-ResBiLSTM-Attention model comprises three parts, including the attention mechanism (AM), the 1D convolutional neural network (1D-CNN), and the residual-bidirectional-long short-term memory (ResBiLSTM).

The integrated deep-learning model, namely the 1DCNN-Residual-BiLSTM-Attention (1DCNN-ResBiLSTM-Attention) model, incorporates three key modules: 1D-CNN, ResBiLSTM, and attention. Initially, the 1D-CNN module is responsible for extracting the local features from the input sequence [34]. The extracted features are then fed into two bidirectional LSTM layers, enhancing the model’s temporal modeling capabilities through residual connections. Finally, the attention module takes the output of the ResBiLSTM module as the input. By computing the weights for each time step, it selectively emphasizes crucial information, thereby improving the model’s expressive capacity. This intricately designed structure facilitates the synergistic operation of different modules, allowing for the comprehensive and accurate capture of multi-level, multi-scale information in the input sequence, ultimately enhancing the overall model performance and generalization ability. The structural diagram is depicted in Figure 1.

### 2.5. Statistical Analysis

In this study, the classification accuracy of each pixel was used as the evaluation metric for the model, as shown in Formula (8) below:

(8) Accuracy=(RGP+MYP)/TNP

where *RGP* is the number of pixels correctly predicted as red ginseng in the image, *MYP* is the number of pixels correctly predicted as mycotoxin in the image, and *TNP* is the total number of pixels in the image excluding the background.

The confusion matrix, known as the error matrix, is a visualization that presents the performance of the algorithm in a specific matrix, where each column represents the predicted category, and each row represents the true category. The corresponding precision, recall rate, and *F*1 score formula are as follows:

(9) Precision=TP/TP+FP)

(10) Recall=TP/(TP+FN)

(11) F1=2∗(Precision∗Recall)/(Precision+Recall)

where *TP* denotes the number of samples labeled as positive and classified as positive, *FP* denotes the number of samples labeled as negative and classified as positive, and *FN* denotes the number of samples labeled as positive and classified as negative.

The system was equipped with an Intel I9-11900K CPU and an NVIDIA GeForce RTX 3090 graphics card with 32 GB of operating memory running on the Windows 10 operating system. The experimental environment was configured with Python 3.8, keras 2.3.1.

## 3. Results and Discussion

### 3.1. Construction of Pixel-Level Mycotoxin Recognition

**Figure 2:**
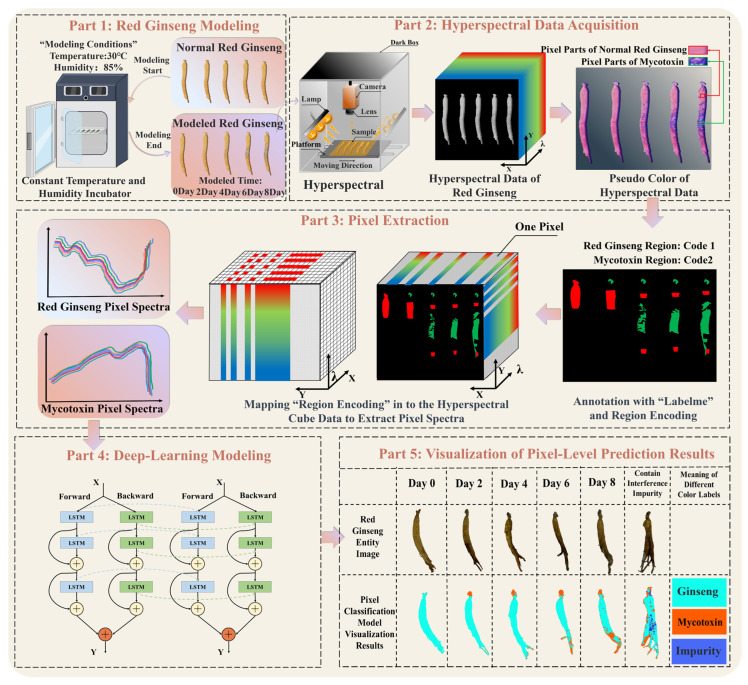
The process of the pixel-level recognition of mycotoxins in red ginseng. The process comprises five parts, including red ginseng modeling, hyperspectral data acquisition, pixel extraction, deep-learning modeling, and the visualization of the pixel-level prediction results.

The overall process of the pixel-level recognition of mycotoxins in red ginseng based on hyperspectral imaging technology combined with deep-learning algorithms is illustrated in Figure 2. The main steps of this study are as follows:

(Ⅰ) Cultivation of moldy red ginseng and collection of HIS;

(II) Extraction of pixels containing mycotoxins and red ginseng and training with the 1DCNN-ResBiLSTM-Attention model;

(III) Addition of interfering substances encountered in the real-world process and storage of red ginseng and training with corresponding pixel spectra in the model;

(IV) Comparison between the trained 1DCNN-ResBiLSTM-Attention model and traditional machine learning models;

(Ⅴ) Pixel-level recognition of real red ginseng samples and visualization of the detection results.

### 3.2. Spectral Curve Analysis

**Figure 3:**
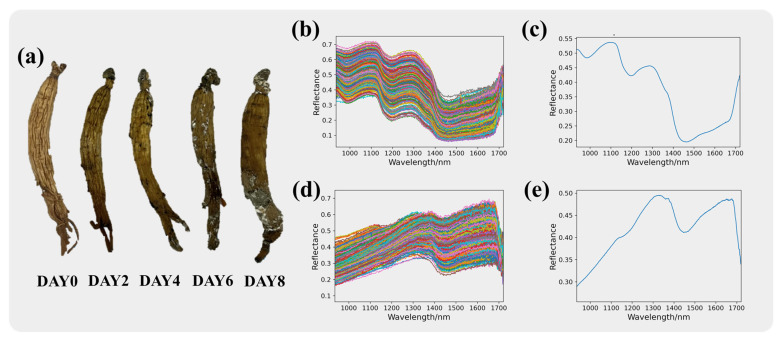
Spectral information of red ginseng. Red ginseng with different molding time is shown in (**a**). The original spectra for red ginseng and the mycotoxins are presented in (**b**) and (**c**), respectively, while the average spectra for red ginseng and the mycotoxins are shown in (**d**) and (**e**), respectively.

Red ginseng after modeling, as shown in Figure 3a, and the original spectral curves of the red ginseng pixels and mycotoxin pixels are shown in Figure 3b and Figure 3c, respectively. The range of the intra-class variance of the samples can be seen in Figure S1. The original spectral curves consist of spectral information from 400 pixels and exhibit noticeable noise. Figure 3d and Figure 3e depict the average spectral curves of the red ginseng pixels and mycotoxin pixels, respectively, which are the mean values of the spectra from 400 pixels. From the spectral curves of the red ginseng pixels, it can be observed that in the near-infrared band, the spectral curve exhibits a downward trend followed by an upward trend. On the other hand, the spectral curves of the mycotoxin pixels show evident dual peaks at 1350 nm and 1650 nm, indicating distinct spectral characteristics.

The significant differences in the reflectance and trends of the red ginseng and mycotoxin pixels indicate noticeable changes in the chemical composition of red ginseng affected by mycotoxin contamination. The prominent differences in the spectral curves can serve as important information for distinguishing between them.

### 3.3. Deep-Learning Model Network Parameter Determination

When designing a deep-learning model, an important consideration is to determine the appropriate network parameters, including the learning rate, number of training epochs, and activation function, as these directly impact the model’s performance and convergence speed. An epoch refers to a complete forward and backward pass of the entire training dataset through the neural network. If the number of training epochs is too large, the model may overfit the training data, resulting in optimization bias and decreased performance on the test dataset. Conversely, if the number of training epochs is too small, the model may not sufficiently learn the data’s features, leading to underfitting. The learning rate (LR) controls the magnitude of parameter updates during each iteration. If the learning rate is too large, parameter updates may exceed a reasonable range, causing the model to miss local optimal solutions and reducing its performance and generalization ability. On the other hand, if the learning rate is too small, parameter updates may be too small, leading to slow convergence and potentially becoming stuck in local optimal solutions or even failing to converge. Activation functions (AFs) play a crucial role in deep learning as they serve as non-linear transformations within neural networks, converting the input of neurons into outputs by introducing non-linear properties. The choice of activation function not only impacts the expressive power and performance of neural networks but also helps to address issues such as vanishing gradients, facilitating better learning and the optimization of the network.

In this study, the R-M dataset was used as an example to determine the optimal parameters for each network by controlling different numbers of epochs and learning rates. The initial learning rate was set to 10^−2^. Five different levels of epochs, including 100, 200, 300, 400, and 500, were used to determine the optimal number of epochs. After determining the epochs, different learning rate values were adjusted to find the optimal learning rate. The Swish activation function was chosen, and the batch size was set to 32, remaining constant during model training. The results for different numbers of epochs and learning rates are shown in Table 1.

From Table 1, it can be observed that smaller network architectures lead to faster model fitting and training speeds. On the other hand, larger network architectures require more time for training one epoch, resulting in relatively slower fitting speeds. However, when the larger models reach their optimal training state, they achieve higher training accuracy. Among the three models, 1D-CNN showed the fastest fitting speed, reaching 100% training accuracy after the first 100 epochs. It also achieved the highest testing accuracy of 96.67%. At 400 epochs, the model’s testing accuracy reached its peak at 0.9833. Continuing training beyond 400 epochs may have caused overfitting, as indicated by the increase in testing loss after 400 epochs. Based on this observation, the optimal number of epochs for 1D-CNN was set to 400; ResBiLSTM exhibited a relatively slower fitting speed. Similar to 1D-CNN, ResBiLSTM reached its optimal state at 400 epochs, achieving a testing accuracy of 0.9875. Therefore, ResBiLSTM’s optimal number of epochs was also set to 400; 1DCNN-ResBiLSTM-Attention, with the deepest network structure among the three models, required the longest training time. After 100 epochs, it achieved a testing accuracy of 93.33%, which was the lowest among the three models. However, at 500 epochs, 1DCNN-ResBiLSTM-Attention converged to its optimal state, reaching a testing accuracy of 99.17%, which was the highest among the three models. Hence, 1DCNN-ResBiLSTM-Attention’s optimal number of epochs was set to 500.

After determining the optimal number of epochs for each model, the next step was to determine the optimal learning rate for each model. As shown in Table 1, when the learning rate was set to 10^−1^, all three models experienced a gradient explosion [35]. During the backpropagation process, gradients are propagated back to the input layer and used to update the weights of each layer. If the learning rate is too large, the weight updates will be substantial, causing the weight values to rapidly increase. This leads to unstable training, where the model’s training and testing accuracy remains unchanged, and the loss value becomes NaN (the loss increases with each iteration and eventually exceeds the range of floating-point representation, resulting in NaN). This indicates that a gradient explosion occurs when the learning rate is set to 10^−1^, and the learning rate needs to be reduced. Deeper networks like 1DCNN-ResBiLSTM-Attention, which have more parameters and complexity compared to 1D-CNN, require relatively larger learning rates to maintain training stability, prevent the vanishing gradient problem, and accelerate convergence. On the other hand, shallower networks like 1D-CNN are more easily trainable with relatively lower learning rates and may be less prone to overfitting. Due to their fewer parameters, a lower learning rate can effectively control the training process. As shown in Table 1, the optimal learning rates for the 1D-CNN and ResBiLSTM models were 10^−4^ and 10^−3^, respectively, where both models achieved higher testing accuracies than when using the initial learning rate. On the other hand, 1DCNN-ResBiLSTM-Attention, being the largest network structure, required a higher learning rate, and among the tested learning rates, 10^−2^ performed the best for 1DCNN-ResBiLSTM-Attention.

After determining the learning rate and number of training epochs, the final activation function needed for the model was determined. In this experiment, after determining the epochs and learning rate, the models were established using the Sigmoid function, Tanh function, and Swish function, respectively. Table 1 shows the results of the models with different activation functions. It can be observed that Swish achieved the best results among the three models, with the 1DCNN-ResBiLSTM-Attention model achieving the highest accuracy of 0.9917. The Swish activation function, proposed by researchers at Google Brain in 2017, possesses advantages such as smoothness, non-monotonicity, and adaptiveness, which helps to enhance the performance of deep neural networks. In contrast, the Tanh and Sigmoid activation functions, although still useful in certain situations, tend to suffer from gradient vanishing problems. Therefore, Swish was selected as the activation function for this study.

In summary, through the settings of various parameters, the optimal training epoch number for the 1D-CNN model was determined to be 400 with a learning rate of 10^−4^, and the Swish activation function was selected. Similarly, the optimal training epoch number for the ResBiLSTM model was also determined to be 400 with a learning rate of 10^−3^, and the Swish activation function was chosen. Finally, the 1DCNN-ResBiLSTM-Attention model achieved the best performance with 500 training epochs, a learning rate of 10^−2^, and the Swish activation function.

### 3.4. The Establishment of the “Red Ginseng-Mycotoxin-Interference Impurity” Three-Classification Model

In “Section 3.2”, we observed that the deep-learning models achieved very high accuracies in identifying the red ginseng and mycotoxin pixels on the R-M dataset. Specifically, the 1DCNN-ResBiLSTM-Attention model achieved a testing accuracy of 99.17% after training for 500 epochs, demonstrating the successful recognition of the red ginseng and mycotoxin pixels using the combination of hyperspectral imaging and deep-learning algorithms. However, in real-world scenarios, mycotoxin recognition is often not a simple binary classification problem. For instance, during the storage of red ginseng, there might be various interference substances on the surface, such as dust and soil, which are common in real-world scenarios. These substances can physically resemble mycotoxins and introduce interference in the recognition process. If we only train the mycotoxin recognition model on the R-M dataset, it may misclassify interference substances as either red ginseng or mycotoxin, leading to significant misjudgments that do not align with real-world requirements.

To address this issue, this study built upon the model established in “Section 3.2” and created the “Red ginseng-Mycotoxin-Interference Impurity” (R-M-I) dataset. This new dataset included interference impurities commonly found in real-world scenarios, such as “dust” and “soil” The pixels of these interference impurities were labeled as “3” using Labelme, representing a third class that was neither red ginseng nor a mycotoxin. This addition aimed to increase the model’s generalization ability and improve its recognition performance on unknown samples. By incorporating the interference impurities, the trained model was better equipped to handle real-world situations, enhancing its feasibility and applicability in practical scenarios.

#### 3.4.1. Spectral Curves of the Interfering Impurities

**Figure 4:**
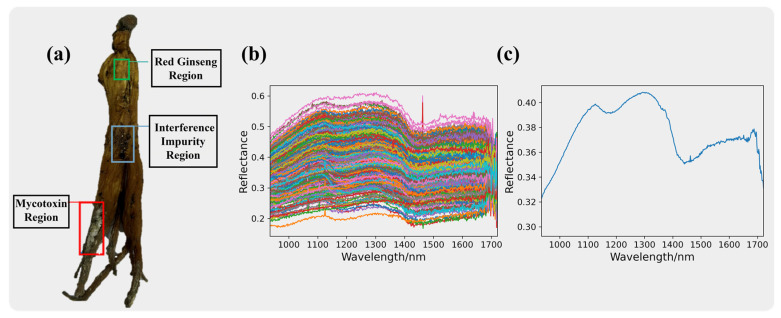
Red ginseng with interfering impurities is shown in (**a**). The spectra information of the interference impurities. The original spectra for the interference impurities are presented in (**b**), while the average spectra for the interference impurities are shown in (**c**).

The pixel spectra of the interference impurities are presented in Figure 4, where Figure 4b displays the original spectra of the interference impurity pixels. The spectra of the interference impurities show severe noise and information redundancy. After averaging the spectra of 400 pixels, the average spectrum of the interference impurities is shown in Figure 4c.

The interference impurities comprised a diverse range of substances, and some of them may have had physical properties and colors similar to mycotoxins, making it challenging to distinguish them using the naked eye or RGB cameras. However, from the spectral curve plots, we can observe significant differences between the interference impurities and mycotoxins. This indicates that hyperspectral imaging technology has unique advantages in identifying two substances with similar appearances.

#### 3.4.2. Comparison of Different Deep-Learning Models

In “3.3”, we determined the optimal training parameters for 1D-CNN, ResBiLSTM, and 1DCNN-ResBiLSTM-Attention. Based on these parameters, we conducted training using the R-M-I dataset. The training results are presented in Table 2.

From Table 2, we can observe that after adding the interference impurities, the number of sample categories increased, and the complexity of the dataset also increased. Consequently, the training difficulty for the models also increased, leading to a slight decrease in the test accuracy compared to the R-M dataset. However, 1DCNN-ResBiLSTM-Attention still performed the best in the R-M-I dataset, achieving a test accuracy of 96.39%. This could be attributed to the larger dataset size, allowing the deeper network structure of 1DCNN-ResBiLSTM-Attention to better fit the data. As a result, 1DCNN-ResBiLSTM-Attention exhibited the best performance in this scenario. On the other hand, the ResBiLSTM and 1D-CNN models showed relatively lower performance, with test accuracies of 94.72% and 93.33%, respectively. Based on the above results, 1DCNN-ResBiLSTM-Attention was selected as the optimal model for identifying mycotoxin pixels in red ginseng.

**Figure 5:**
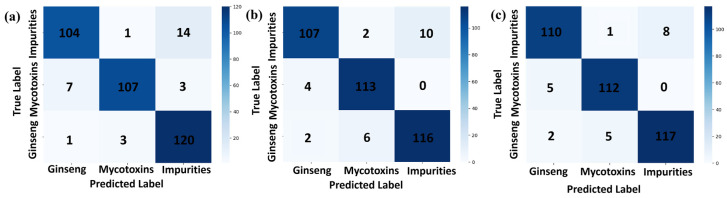
Confusion matrix results of the three-class classification models ((**a**): 1D-CNN, (**b**): ResBiLSTM, (**c**): 1DCNN-ResBiLSTM-Attention).

The confusion matrix is a tool for representing the performance of a classifier in the matrix form, also known as an error matrix. Each row of the confusion matrix represents the true class, while each column represents the classifier’s prediction, as shown in Figure 5, where Figure 5a represents 1D-CNN, Figure 5b represents ResBiLSTM, and Figure 5c represents 1DCNN-ResBiLSTM-Attention, and the precision, recall, and F1 scores are shown in Table 3. From the figure, it can be seen that the 1DCNN-ResBiLSTM-Attention model exhibited the best classification ability in distinguishing the red ginseng pixels, mycotoxin pixels, and interference impurities and had the highest accuracy among all the models. The 1D-CNN model performed the worst among all the models, showing the most misclassifications. Across the three models, the recognition accuracy of the interference impurity pixels was highest during the recognition process of the three classes of targets, while the misclassification rate of the mycotoxin pixels was the highest.

### 3.5. Comparison with Traditional Machine Learning Models

In the field of hyperspectral imaging, traditional machine learning algorithms are applied earlier and are more widely used. The typical processing workflow when using traditional machine learning models includes the following steps: (1) Data collection and ROI determination: This step involves collecting data and determining the ROIs in the images. (2) Spectral data pre-processing: Pre-processing steps, such as band selection, denoising, and data standardization, are performed on the spectral data. (3) Data splitting: The prepared dataset is split into training and testing sets. The training set is used to train the model, while the testing set is used to evaluate the model’s performance. (4) Model selection: In traditional machine learning, choosing an appropriate model is crucial. Common traditional machine learning models include partial least squares-discriminant analysis (PLS-DA), support vector machine (SVM), random forest (RF), K-nearest neighbors (KNNs), and others.

In this study, five pre-processing methods (first-order derivative (D1), second-order derivative (D2), SG smoothing, multiplicative scatter correction (MSC), and standard normal variate transform (SNV)) were combined with four classification models (PLS-DA, SVM, RF, and KNN) for each possible combination. The performance was evaluated using 10-fold cross-validation on the classification accuracies for the training, validation, and testing sets. The results are shown in Table 4. The results indicate that after pre-processing, the accuracy of the models generally improved, with the MSC combined with RF achieving the best performance, reaching a test accuracy of 94.87%. However, compared to 1DCNN-ResBiLSTM-Attention, the SVM model’s accuracy was still relatively lower, and it required selecting suitable pre-processing methods. On the other hand, the 1DCNN-ResBiLSTM-Attention model used in this study achieved higher accuracy without the need for manual pre-processing and feature band selection, making it easier to train and yield superior results.

**Figure 6:**
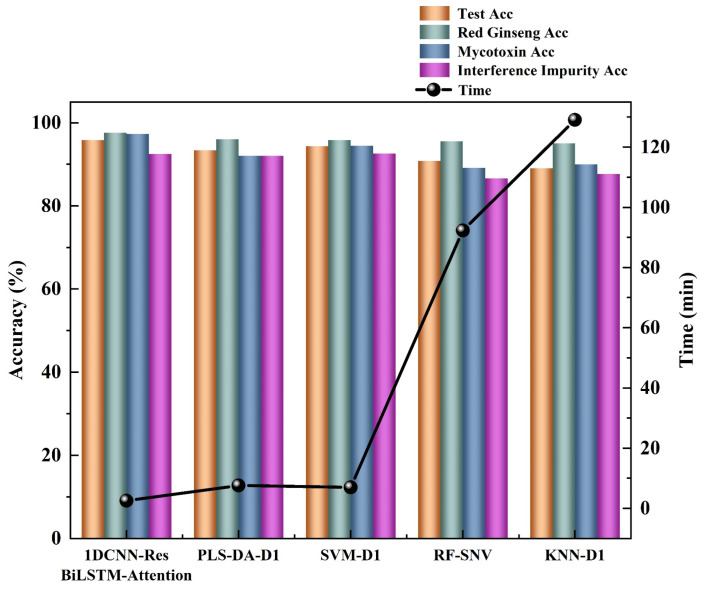
Plot of the accuracies of each category for different models and prediction times for different models. The height of the bar chart represents the accuracy, while the black circles represent the time required for model building.

In addition to comparing the testing accuracy, this study also compared the prediction time of the trained models. Although deep learning requires a longer training time compared to traditional machine learning due to the complexity of its networks, it benefits from hardware acceleration such as GPUs, resulting in a faster prediction speed and better meeting the real-time detection requirements in the field. The results of importing a hyperspectral image of size 829 (X) * 640 (Y) * 512 (λ) into different models and comparing the prediction time are shown in Figure 6. It can be observed that there is a significant difference in the prediction times among different models. The deep-learning models had the fastest prediction speed, with 1DCNN-ResBiLSTM-Attention only requiring 2.5 min to predict the entire hyperspectral image. On the other hand, traditional machine learning models had much slower prediction speeds. The SVM model was the fastest among the traditional machine learning models, but it still took 7.6 min to predict the hyperspectral data. The KNN model was the slowest among all the models, requiring 129.08 min to predict a single hyperspectral image. The above results are attributed to the following reasons: (1) Deep-learning models typically involve a significant amount of matrix computations, which can be efficiently processed in parallel on GPUs. In contrast, traditional machine learning models may require more serial computations, resulting in the underutilization of GPU’s parallel computing capabilities. (2) Deep-learning frameworks such as PyTorch and TensorFlow are highly optimized for GPUs, leading to increased efficiency when running deep-learning models on GPUs. Additionally, specialized hardware designed for deep-learning tasks, such as GPUs and TPUs, can be utilized to accelerate computations, whereas traditional machine learning models usually do not fully exploit hardware accelerators. These results demonstrate that deep-learning models, especially 1DCNN-ResBiLSTM-Attention, offer significantly faster prediction times compared to traditional machine learning models. This advantage is valuable in practical applications where real-time or near real-time predictions are required.

In conclusion, compared to traditional machine learning models, deep learning is capable of handling complex non-linear problems and highly abstract tasks, leading to higher prediction accuracy. Moreover, it can fully leverage the parallel computing capabilities of hardware accelerators such as GPUs and TPUs, significantly speeding up the training and inference processes. In practical mycotoxin detection, deep-learning models offer greater advantages and practicalities.

### 3.6. Feature Band Selecting Based on the Attention Mechanism

**Figure 7:**
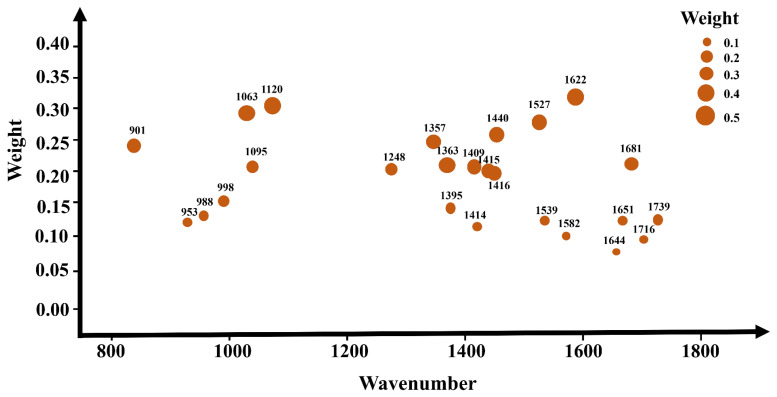
The importance levels of different spectral bands based on the attention mechanism. The different circles in the graph represent different feature wavelengths, with larger circles indicating higher corresponding weights.

Attention mechanisms are widely applied in deep learning for the purpose of feature selection, simulating the human attention mechanism to enable models to focus on the most relevant information for a given task when processing input data. In attention mechanisms, models learn weight allocations, allowing different parts of the input to be assigned varying degrees of attention for a specific task. Concerning the selection of important features, attention mechanisms empower models to dynamically adjust the weights of features based on the input data context, concentrating more on information beneficial to the task. This mechanism enables models to adapt more flexibly to different input scenarios and demonstrates superior performance when dealing with tasks where the importance of features varies. In this study, attention mechanisms were employed to select important spectral bands, as illustrated in Figure 7. Figure 7 illustrates the weights obtained for different wavelengths through an attention mechanism after the model is established. A higher weight for a wavelength indicates its greater importance during model building. Table 5 and Figure S2 present the accuracy results and confusion matrix results of various models constructed based on the feature band selection attention mechanism, where Figure S2a represents 1D-CNN, Figure S2b represents ResBiLSTM, and Figure S2c represents 1DCNN-ResBiLSTM-Attention and the precision, recall, and F1 scores are shown in Table 6. 

In the task of discriminating between the red ginseng, mycotoxins and interfering impurities, the application of the attention mechanism for extracting important spectral bands led to the highest accuracy on the test set for the 1DCNN-ResBiLSTM-Attention model, reaching 96.94%, and the confusion matrix results also indicate that the 1DCNN-ResBiLSTM-Attention model achieved the highest recognition rate for the three classes of target pixels. Compared to the full-wavelength model, the performance of the model constructed using only the top 25 feature bands extracted using the AM already surpassed the accuracy of the full-wavelength model, demonstrating the advantage of using the attention mechanism to select feature bands. Both the 1D-CNN and ResBiLSTM-Attention models also exhibited comparable performance to the full-wavelength model. Moreover, the application of the attention mechanism for feature band selection resulted in a significant reduction in data volume, leading to a notable improvement in the model computation speed. The training time for 1DCNN-ResBiLSTM-Attention, initially 2.1 h, was reduced to 0.37 h, representing only one-tenth of the original modeling time for the entire wavelength range. These results highlight the significant impact of the attention mechanism in feature band selection, accompanied by a substantial reduction in computational costs.

### 3.7. Visualization of the Pixel-Level Predictions for Unknown Samples

**Figure 8:**
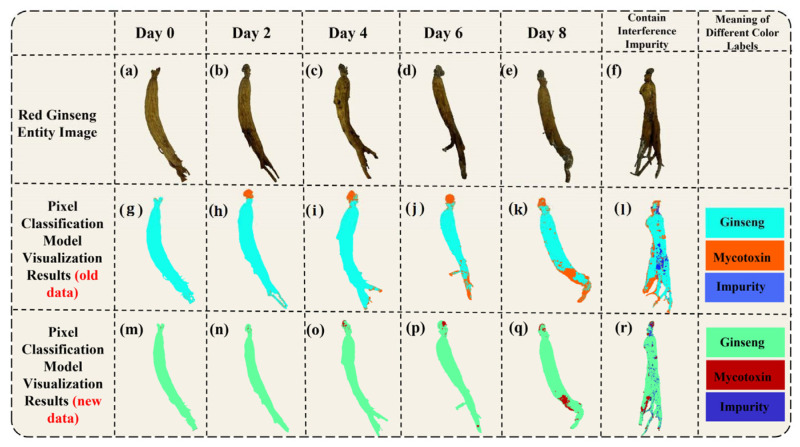
Visualization of the pixel-level prediction results (**a**–**f**) are the RGB images of moldy red ginseng, (**g**–**l**) are the pseudo-color images synthesized using three bands of hyperspectral data, and (**m**–**r**) are the model’s prediction results (the green pixels represent the predicted red ginseng, the red pixels represent the predicted mycotoxins, and the blue pixels represent the predicted interference impurities)).

To visualize the predictions of the 1DCNN-ResBiLSTM-Attention model on the red ginseng spectral images, we first extracted all the pixels from the red ginseng hyperspectral data and imported their spectral information into the trained best-performing 1DCNN-ResBiLSTM-Attention model. This process yielded the prediction results for each pixel. Then, we created RGB images corresponding to the extracted spectra and generated grayscale images from them. Next, we assigned the predicted results of each pixel to their corresponding positions in the grayscale image. Finally, we converted the grayscale image into a pseudo-color image based on the differences in the predictions for each pixel. The resulting visualization provides a per-pixel view of the red ginseng classification. The visualized results are displayed in Figure 8.

Figure 8a–f shows the physical images of the red ginseng samples, where Figure 8a–e represents the samples cultured on different days, and Figure 8f represents a red ginseng sample with added interference. Figure 8g–l displays pseudo-color images generated from the high-spectral data. In the pseudo-color images, the green region represents the normal red ginseng area, the dark pink region represents the area with mycotoxins, and the black region in Figure 8l represents the area with added interference. Figure 8m–r depicts the corresponding visualized results of the model’s predictions for the red ginseng samples. In the visualized results, light blue indicates the predicted red ginseng pixels, orange indicates the predicted mycotoxin pixels, and dark blue indicates the predicted interference pixels.

The color distribution in the visualization results shows that the vast majority of the pixels were correctly classified, and even the low-content regions of the true mycotoxin (the lighter-colored parts in Figure 8g–l) can be identified. However, there was a higher probability of misclassification at the edges of the red ginseng sample. There are several potential reasons for this: (1) Inaccurate labeling using Labelme: The pixels at the edges of the red ginseng samples might have been mislabeled as either red ginseng or true mycotoxin pixels during the annotation process, leading to incorrect model predictions. (2) Differences in thickness and content at the edges: The edge regions of the red ginseng samples may have had different thicknesses and contents compared to the central regions, which could have introduced some interference in the prediction results. To improve the accuracy of the predictions at the edges, it may be necessary to carefully review and correct the labeling process and ensure that the training dataset represents a diverse range of red ginseng samples, including variations in thickness and content at different regions. 

Pixel-level recognition offers multiple advantages: (1) Each pixel can serve as a training sample, which significantly expands the dataset size without increasing the number of experimental samples. This enlargement of the dataset enhances the model’s generalization capability and robustness. (2) By training the model on pixel-level data, predictions can be made at the pixel level, resulting in more detailed and precise detection results, leading to higher accuracy in the detection process.

In the detection of mycotoxins in TCMs, ELISA has advantages such as high accuracy and strong stability. However, this method has a long detection cycle and may lead to sample destruction. When using RGB cameras or industrial cameras for mycotoxin detection, there are limitations, such as difficulty in detecting low levels of mycotoxins in the early stages and susceptibility to interference from impurities with similar colors. Nevertheless, HSI can achieve high accuracy by distinguishing differences in spectral curves, even in cases of low mycotoxin content and the presence of interfering impurities, effectively solving the problems associated with the aforementioned technologies.

At present, there have been no reports on the detection of mycotoxins in traditional Chinese medicine using HSI, but there are more reports in agricultural fields such as peanuts and corn. Gao et al. [36] conducted mold modeling on corn by dripping an aflatoxin B1 solution and collecting hyperspectral data. They employed three different methods for feature band extraction and combined them with random forest (RF) and K-nearest neighbor (KNN) algorithms for the pixel-level identification of aflatoxin B1 in corn. The results indicated that using Relieff for feature extraction combined with the KNN classifier achieved a pixel-level recognition accuracy of 98.77% for aflatoxin B1 in corn. Han et al. [28] utilized hyperspectral data collected from aflatoxin B1-dripped peanuts and designed a 1D-CNN network for pixel-level identification. Their findings demonstrated that the 1D-CNN achieved a recognition accuracy of 96% for aflatoxin B1 in peanuts, surpassing traditional machine learning methods. Liu et al. [29] collected hyperspectral data from moldy peanuts and employed data feature pre-extraction along with the multi-feature (MF) block fusion technique combined with the Hypernet model for the pixel-level identification of moldy peanuts. The experimental results revealed that the Hypernet-PRMF model achieved an average accuracy of 92.07%.

Although the accuracy of detecting aflatoxin B1 standard samples using hyperspectral pixel-level detection technology is high, this study also achieved a high accuracy in detecting real moldy samples. Even with the addition of interfering impurities, the accuracy still reached 96.11%. Even though Liu [29] successfully conducted the pixel-level detection of moldy peanuts using hyperspectral imaging, the interference commonly encountered in real-life detection scenarios was not considered. Compared to previous studies focusing on the pixel-level detection of mycotoxins using hyperspectral imaging, this research not only successfully conducted the pixel-level detection of real-life moldy ginseng, achieving an accuracy of 96.11% but also examined common interfering impurities present in real-life detection scenarios, thereby enhancing the model’s practicality. Lastly, through attention mechanisms, important feature bands in mycotoxin pixel-level detection were explored, and GPU hardware acceleration was utilized to shorten the time consumed for online detection, making it more suitable for real-time field detection.

In conclusion, the recognition of mycotoxins based on hyperspectral imaging is feasible, and the visualization maps provide an intuitive display of the prediction results for each pixel. This approach offers a practical and viable method for mycotoxin recognition. Compared to traditional machine learning models, deep-learning models exhibit significant advantages in handling complex non-linear problems and abstract tasks. The findings of this study provide an effective solution for accurately identifying mycotoxin pixels in red ginseng, offering strong support for quality assessment and safety assurance.

## 4. Conclusions

This study proposes the use of HSI for the pixel-level recognition of mycotoxins in ginseng. Firstly, the HSI of ginseng with different degrees of mold was collected. The ROI containing ginseng pixels and mycotoxin pixels was extracted using background segmentation and encoded for position information to construct the dataset. Based on the spectral differences between ginseng and the mycotoxins, a pixel-level classification was performed. After adjusting the parameters of the deep-learning model, the test accuracy of 1D-CNN, ResBiLSTM, and 1DCNN-ResBiLSTM-Attention on the R-M dataset reached 98.75%, 99.03%, and 99.17%, respectively, demonstrating the reliable recognition capability of HSI for ginseng and mycotoxin pixels.

To better simulate real-world scenarios and enhance the model’s practicality, we introduced an R-M-I dataset with potential interferences during detection and trained it using the different deep-learning models. During training, the 1DCNN-ResBiLSTM-Attention model was selected with a learning rate of 10^−2^ and 500 epochs to determine the network structure. The results showed that the 1DCNN-ResBiLSTM-Attention model based on HSI achieved the highly accurate and speedy recognition of ginseng and the mycotoxins, as well as the interfering impurities. For the R-M-I dataset, the 1DCNN-ResBiLSTM-Attention model achieved a test accuracy of 96.39% and successfully performed pixel-level predictions on unknown samples. Compared to traditional machine learning models, it exhibited superior detection accuracy and speed.

In conclusion, this study successfully applied HSI for the pixel-level recognition of mycotoxins in ginseng and visually presented the prediction results for each pixel. This method provides a practical approach for mycotoxin recognition and represents the first application in the field of TCM, contributing to the intelligent analysis of herbal medicine with significant implications. This technology can serve as upstream detection for online sorting machines of TCMs and can be combined with mechanical arms to screen out moldy medicinal materials that do not meet the standards. This study only qualitatively identified mycotoxins in different degrees of moldy ginseng without predicting the actual content of mycotoxins. Future research should focus on determining the chemical composition of the mycotoxins in ginseng and establishing quantitative models using hyperspectral imaging. Deep-learning algorithms should be utilized for the real-time measurement of mycotoxin content in ginseng.

## Figures and Tables

**Table 1:** Accuracy of each item under different epochs and LRs.

Model	Parameter (Epoch)	Train Accuracy	Train Loss	Test Accuracy	Test Loss	Parameter (LR)	Train Accuracy	Train Loss	Test Accuracy	Test Loss	Parameter (AF)	Train Accuracy	Train Loss	Test Accuracy	Test Loss
1D-CNN	100	1.0000	0.0134	0.9667	0.0808	10^−1^	0.4898	Nan	0.5250	Nan	Sigmoid	1.0000	0.0482	0.9708	0.0812
	200	1.0000	0.0038	0.9750	0.0804	10^−2^	1.0000	0.0002	0.9833	0.0698	Tanh	0.9893	0.0345	0.9625	0.1165
	300	1.0000	0.0014	0.9792	0.0799	10^−3^	1.0000	0.0002	0.9833	0.0480	Swish	1.0000	0.0171	0.9875	0.0564
	400	1.0000	0.0002	0.9833	0.0698	10^−4^	1.0000	0.0171	0.9875	0.0564	-	-	-	-	-
	500	1.0000	0.0001	0.9833	0.1032	10^−5^	0.9679	0.1203	0.9708	0.1017	-	-	-	-	-
ResBiLSTM	100	0.9536	0.1578	0.9458	0.1447	10^−1^	0.4893	Nan	0.5250	Nan	Sigmoid	0.9964	0.0134	0.9833	0.0730
	200	0.9732	0.0991	0.9708	0.0831	10^−2^	1.0000	0.0001	0.9875	0.0866	Tanh	0.9911	0.0135	0.9667	0.0911
	300	0.9929	0.0204	0.9792	0.1663	10^−3^	1.0000	0.0003	0.9903	0.0419	Swish	1.0000	0.0003	0.9903	0.0419
	400	1.0000	0.0001	0.9875	0.0866	10^−4^	0.9964	0.0195	0.9875	0.0534	-	-	-	-	-
	500	0.9964	0.0037	0.9875	0.1185	10^−5^	0.9804	0.0980	0.9708	0.1159	-	-	-	-	-
1DCNN-ResBiLSTM-Attention	100	0.9554	0.1351	0.9333	0.2678	10^−1^	0.4893	Nan	0.5250	Nan	Sigmoid	1.0000	0.0052	0.9833	0.1343
	200	0.9696	0.0793	0.9542	0.1323	10^−2^	1.0000	0.0001	0.9917	0.0879	Tanh	0.9982	0.0049	0.9833	0.1084
	300	0.9832	0.0580	0.9780	0.0919	10^−3^	1.0000	0.0002	0.9833	0.1059	Swish	1.0000	0.0001	0.9917	0.0879
	400	1.0000	0.0002	0.9833	0.0847	10^−4^	1.0000	0.0023	0.9792	0.0656	-	-	-	-	-
	500	1.0000	0.0001	0.9917	0.0879	10^−5^	0.9857	0.0743	0.9750	0.0875	-	-	-	-	-

Note: The loss increases with each iteration and eventually exceeds the range of floating-point representation, resulting in NaN.

**Table 2:** Various accuracy rates for different deep-learning models (in R-M dataset).

	Evaluation Index	Train Accuracy	Train Loss	Test Accuracy	Test Loss	Time (h)
Model						
1D-CNN		0.9695	0.1107	0.9333	0.2626	0.52
ResBiLSTM		0.9821	0.0686	0.9472	0.2008	1.7
1DCNN-ResBiLSTM-Attention		1.0000	0.0133	0.9639	0.1689	2.1

**Table 3:** Precision, recall, and F1 scores for different deep-learning models.

Model	Class	Precision	Recall	F1 Score
1D-CNN	Red ginseng	0.9391	0.9076	0.9231
	Mycotoxin	0.9417	0.9658	0.9536
	Interfering impurities	0.9200	0.9274	0.9237
ResBiLSTM	Red ginseng	0.9252	0.9328	0.9289
	Mycotoxin	0.9739	0.9573	0.9655
	Interfering impurities	0.9440	0.9516	0.9478
1DCNN-ResBiLSTM-Attention	Red ginseng	0.9565	0.9244	0.9402
	Mycotoxin	0.9741	0.9658	0.9700
	Interfering impurities	0.9380	0.9758	0.9565

**Table 4:** Classification accuracy of the models with different pre-processing methods.

Model	Pre-Processing	Train Accuracy	Test Accuracy
PLS-DA	-	0.9264	0.9187
	D1	0.9820	0.9359
	D2	0.9637	0.9215
	SG	0.9124	0.9076
	SNV	0.9272	0.8921
	MSC	0.5995	0.5929
SVM	-	0.9383	0.9235
	D1	0.9848	0.9429
	D2	0.9561	0.9174
	SG	0.9279	0.9172
	SNV	0.9701	0.9449
	MSC	0.6687	0.6273
RF	-	0.9976	0.9177
	D1	0.9945	0.9404
	D2	0.9874	0.8871
	SG	0.9973	0.9171
	SNV	0.9943	0.9414
	MSC	0.9974	0.9487
KNN	-	0.9189	0.9096
	D1	0.9553	0.9472
	D2	0.9302	0.9096
	SG	0.9185	0.9091
	SNV	0.9297	0.8907
	MSC	0.9173	0.8644

**Table 5:** Various accuracy rates for different deep-learning models (in R-M-I dataset).

	Evaluation Index	Train Accuracy	Train Loss	Test Accuracy	Test Loss	Time (h)
Model						
1D-CNN		0.9762	0.2404	0.9278	0.2697	0.06
ResBiLSTM- Attention		0.9833	0.1464	0.9417	0.1953	0.18
1DCNN-ResBiLSTM-Attention		1.0000	0.0729	0.9694	0.1763	0.37

**Table 6:** Precision, recall, and F1 scores for different deep-learning models (after feature selection).

Model	Class	Precision	Recall	F1 Score
1D-CNN	Red ginseng	0.9024	0.9328	0.9174
	Mycotoxin	0.9333	0.9573	0.9451
	Interfering impurities	0.9487	0.8952	0.9212
ResBiLSTM	Red ginseng	0.9727	0.8892	0.9345
	Mycotoxin	0.9569	0.9487	0.9528
	Interfering impurities	0.9030	0.9758	0.9380
1DCNN-ResBiLSTM-Attention	Red ginseng	0.9741	0.9496	0.9617
	Mycotoxin	0.9744	0.9744	0.9744
	Interfering impurities	0.9606	0.9837	0.9721

## Data Availability

The original data presented in the study are openly available at https://doi.org/10.5281/zenodo.11082600 https://zenodo.org/records/11082600. In the Zip, spectral. npy was the average spectral data of red ginseng, mycotoxins, and interference impurities, and label. npy was the corresponding label. The spectral data format was [1200,510] and the label data format was [1200,1]. For specific operations, see https://zenodo.org/records/11082600.

## Supplementary Materials

The following supporting information can be downloaded at: https://www.mdpi.com/article/10.3390/s24113457/s1, Figure S1: The average spectral and standard deviation; Figure S2: The confusion matrix results of the three-class classification model after feature extraction; Table S1: Experimental equipments; Table S2: Molecular vibration attribution table.

## References

[1] Wang C.-Z., Anderson S., Du W., He T.-C., Yuan C.-S. (2016). Red ginseng and cancer treatment. Chin. J. Nat. Med..

[2] Yang M.C., Seo D.S., Hong J.K., Hong S.H., Kim Y.C., Lee K.R. (2008). Ginsenosides from the roots of Korean cultivated-wild ginseng. Nat. Prod. Sci..

[3] Zhang L., Dou X.-W., Zhang C., Logrieco A.F., Yang M.-H. (2018). A review of current methods for analysis of mycotoxins in herbal medicines. Toxins.

[4] Ałtyn I., Twarużek M. (2020). Mycotoxin contamination concerns of herbs and medicinal plants. Toxins.

[5] D’Ovidio K., Trucksess M., Weaver C., Horn E., Mcintosh M., Bean G. (2006). Aflatoxins in ginseng roots. Food Addit. Contam..

[6] Trucksess M., Weaver C., Oles C., D’Ovidio K., Rader J. (2006). Determination of aflatoxins and ochratoxin A in ginseng and other botanical roots by immunoaffinity column cleanup and liquid chromatography with fluorescence detection. J. AOAC Int..

[7] Trucksess M.W., Weaver C.M., Oles C.J., Fry F.S., Noonan G.O., Betz J.M., Rader J.I. (2019). Determination of Aflatoxins B1, B2, G1, and G2 and Ochratoxin A in Ginseng and Ginger by Multitoxin Immunoaffinity Column Cleanup and Liquid Chromatographic Quantitation: Collaborative Study. J. AOAC Int..

[8] Qin L., Jiang J.-Y., Zhang L., Dou X.-W., Ouyang Z., Wan L., Yang M.-H. (2020). Occurrence and analysis of mycotoxins in domestic Chinese herbal medicines. Mycol.—Int. J. Fungal Biol..

[9] Trucksess M.W., Scott P.M. (2008). Mycotoxins in botanicals and dried fruits: A review. Food Addit. Contam. Part A—Chem. Anal. Control Expo. Risk Assess..

[10] Kuang Y., Qiu F., Kong W., Luo J., Cheng H., Yang M. (2013). Simultaneous quantification of mycotoxins and pesticide residues in ginseng with one-step extraction using ultra-high performance liquid chromatography-electrospray ionization tandem mass spectrometry. J. Chromatogr. B—Anal. Technol. Biomed. Life Sci..

[11] Bi B., Bao J., Xi G., Xu Y., Zhang L. (2018). Determination of multiple mycotoxin residues in Panax ginseng using simultaneous UPLC-ESI-MS/MS. J. Food Saf..

[12] Ostry V., Malir F., Toman J., Grosse Y. (2017). Mycotoxins as human carcinogens-the IARC Monographs classification. Mycotoxin Res..

[13] Zhang L., Dou X., Kong W., Liu C., Han X., Yang M. (2017). Assessment of critical points and development of a practical strategy to extend the applicable scope of immunoaffinity column cleanup for aflatoxin detection in medicinal herbs. J. Chromatogr. A.

[14] Romagnoli B., Menna V., Gruppioni N., Bergamini C. (2007). Aflatoxins in spices, aromatic herbs, herb-teas and medicinal plants marketed in Italy. Food Control.

[15] Santos L., Marin S., Sanchis V., Ramos A.J. (2009). Screening of mycotoxin multicontamination in medicinal and aromatic herbs sampled in Spain. J. Sci. Food Agric..

[16] Wei R., Qiu F., Kong W., Wei J., Yang M., Luo Z., Qin J., Ma X. (2013). Co-occurrence of aflatoxin B1, B2, G1, G2 and ochrotoxin A in Glycyrrhiza uralensis analyzed by HPLC-MS/MS. Food Control.

[17] Pan Y., Zhang H., Chen Y., Gong X., Yan J., Zhang H. (2023). Applications of Hyperspectral Imaging Technology Combined with Machine Learning in Quality Control of Traditional Chinese Medicine from the Perspective of Artificial Intelligence: A Review. Crit. Rev. Anal. Chem..

[18] Liu L.M., Qu H.B. (2020). Recent advancement of chemical imaging in pharmaceutical quality control: From final product testing to industrial utilization. J. Innov. Opt. Health Sci..

[19] Ru C., Li Z., Tang R. (2019). A Hyperspectral Imaging Approach for Classifying Geographical Origins of Rhizoma Atractylodis Macrocephalae Using the Fusion of Spectrum-Image in VNIR and SWIR Ranges (VNIR-SWIR-FuSI). Sensors.

[20] Zhang J., Xu B., Wang Z., Cheng F. (2022). Application of hyperspectral imaging in the detection of aflatoxin B1 on corn seed. J. Food Meas. Charact..

[21] Pereira da Conceicao R.R., Ferreira Simeone M.L., Vieira Queiroz V.A., de Medeiros E.P., de Araujo J.B., Coutinho W.M., da Silva D.D., Miguel R.d.A., de Paula Lana U.G., de Resende Stoianoff M.A. (2021). Application of near-infrared hyperspectral (NIR) images combined with multivariate image analysis in the differentiation of two mycotoxicogenic Fusarium species associated with maize. Food Chem..

[22] Tao Y., Bao J., Liu Q., Liu L., Zhu J. (2023). Deep residual network enabled smart hyperspectral image analysis and its application to monitoring moisture, size distribution and contents of four bioactive compounds of granules in the fluid-bed granulation process of Guanxinning tablets. Spectrochim. Acta Part A.

[23] Zhang H.X., Pan Y.X., Liu X.Y., Chen Y., Gong X., Zhu J., Yan J., Zhang H. (2023). Recognition of the rhizome of red ginseng based on spectral-image dual-scale digital information combined with intelligent algorithms. Spectrochim. Acta. Part A Mol. Biomol. Spectrosc..

[24] Wang Y., Wang S., Bai R., Li X., Yuan Y., Nan T., Kang C., Yang J., Huang L. (2024). Prediction performance and reliability evaluation of three ginsenosides in Panax ginseng using hyperspectral imaging combined with a novel ensemble chemometric model. Food Chem..

[25] Wang Y., Xiong F., Zhang Y., Wang S., Yuan Y., Lu C., Nie J., Nan T., Yang B., Huang L. (2023). Application of hyperspectral imaging assisted with integrated deep learning approaches in identifying geographical origins and predicting nutrient contents of Coix seeds. Food Chem..

[26] Zhong Y., Ru C.L., Wang S.F., Li Z.H., Cheng Y.Y. (2022). An online, non-destructive method for simultaneously detecting chemical, biological, and physical properties of herbal injections using hyperspectral imaging with artificial intelligence. Spectrochim. Acta Part A—Mol. Biomol. Spectrosc..

[27] Gao J., Zhao L., Li J., Deng L., Ni J., Han Z. (2021). Aflatoxin rapid detection based on hyperspectral with 1D-convolution neural network in the pixel level. Food Chem.

[28] Han Z., Gao J. (2019). Pixel-level aflatoxin detecting based on deep learning and hyperspectral imaging. Comput. Electron. Agric..

[29] Liu Z., Jiang J., Qiao X., Qi X., Pan Y., Pan X. (2020). Using convolution neural network and hyperspectral image to identify moldy peanut kernels. LWT.

[30] Yin Y., Hao Y., Yu H., Liu Y., Hao F. (2017). Detection Potential of Multi-Features Representation of E-Nose Data in Classification of Moldy Maize Samples. Food Bioprocess Technol..

[31] ElMasry G., Wang N., Vigneault C. (2009). Detecting chilling injury in Red Delicious apple using hyperspectral imaging and neural networks. Postharvest Biol. Technol..

[32] Yu J.X., Tang S., Zhangzhong L.L., Zheng W.G., Wang L., Wong A., Xu L.L. (2020). A Deep Learning Approach for Multi-Depth Soil Water Content Prediction in Summer Maize Growth Period. IEEE Access.

[33] Cho K., Courville A., Bengio Y. (2015). Describing Multimedia Content Using Attention-Based Encoder-Decoder Networks. IEEE Trans. Multimed..

[34] Zhang J.J., Liu Y.H., Yuan H. (2023). Attention-Based Residual BiLSTM Networks for Human Activity Recognition. IEEE Access.

[35] Liu M., Chen L., Du X., Jin L., Shang M. (2023). Activated Gradients for Deep Neural Networks. IEEE Trans. Neural Netw. Learn. Syst..

[36] Gao J.Y., Ni J.G., Wang D.W., Deng L.M., Li J., Han Z.Z. (2020). Pixel-level aflatoxin detecting in maize based on feature selection and hyperspectral imaging. Spectrochim. Acta Part A—Mol. Biomol. Spectrosc..

