# Supplementary material for: Pixel-Level Recognition of Trace Mycotoxins in Red Ginseng Based on Hyperspectral Imaging Combined with 1DCNN-Residual-BiLSTM-Attention Model
Source: Sensors (Basel). 2024 May 27;24(11):3457. doi: 10.3390/s24113457 (PMC11174722; doi:10.3390/s24113457)
Supplement: Supplementary file 1 [file sensors-24-03457-s001.zip › sensors-2950505-supplementary.pdf]

# Supplementary Materials

## Tables

**Table S1. Experimental equipments**

| Equipment Name | Model number | Manufacturer |
|---|---|---|
| Halogen lamp | 3900 ER | Illumination Technologies Inc., Boxborough, MA, USA |
| Horizontal moving platform | ETH 14 | Foshan, China |
| Image Acquisition Software | Spectral Image | Xinzhu, China |
| Near-infrared spectral camera | OWL-640-mini | Raptor, Belfast, Northern Ireland, UK |
| Near Infrared Focusing Lens | OLES 23 | Specim, Oulu, Finland |
| Imaging spectrometer | ImSpector-V 10E | Specim, Oulu, Finland |
| Constant temperature and humidity incubator | HPP 750 | Memmert, Germany |

**Table S2. Molecular vibration attribution table**

| Band (nm) | Class | Molecular vibrations |
|---|---|---|
| 1063-1120 | Red ginseng | The third stretching overtone region of -O–H |
| 1357-1440 | Mycotoxins | The first overtone region of free O-H |
| 1622 | Red ginseng | The first overtone region of C-H stretching vibrations |

# Figures

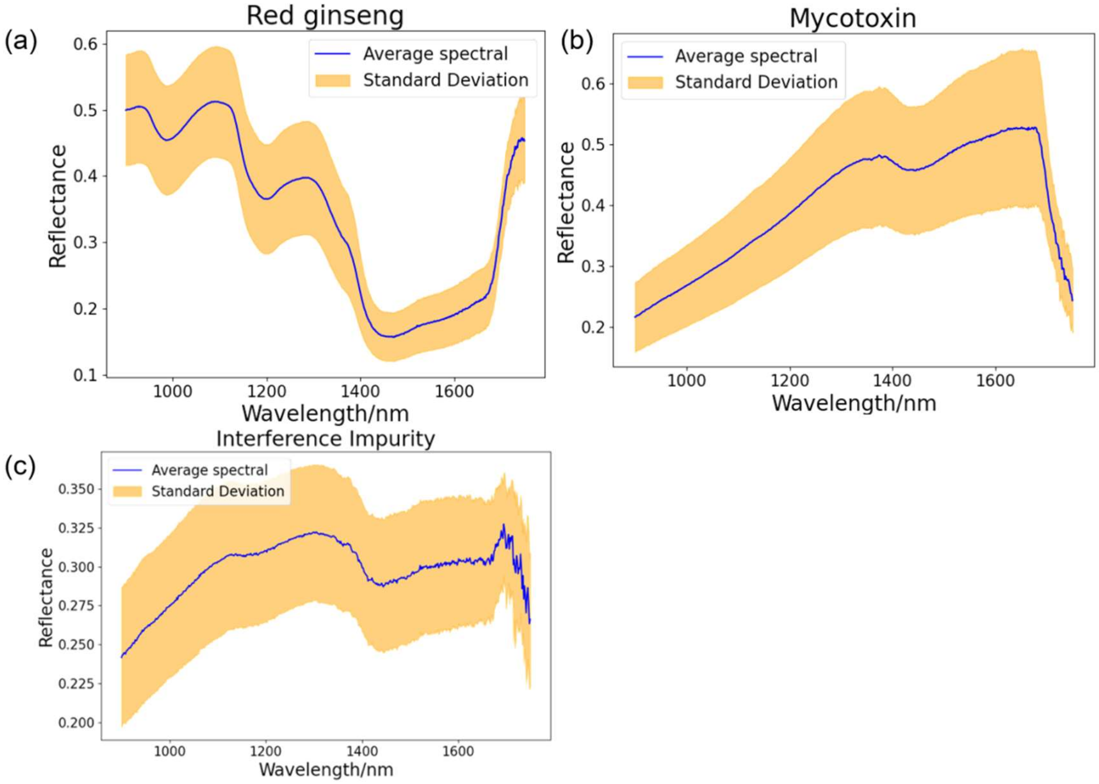


**Figure S1 The average spectral and standard deviation**

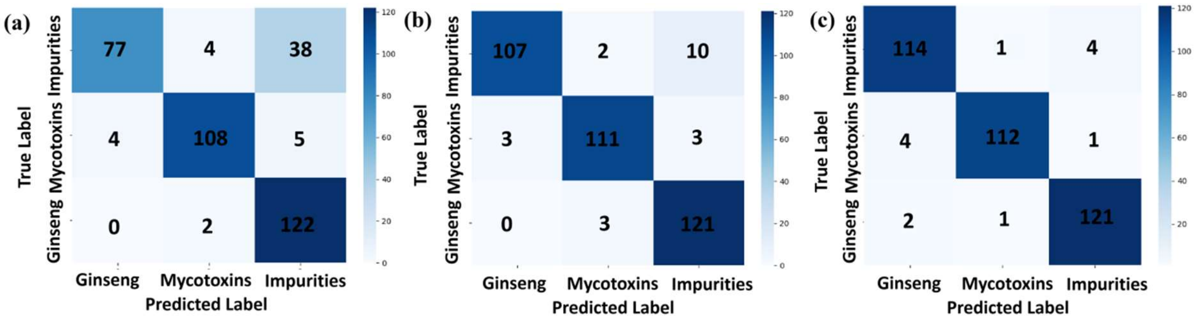


**Figure S2. The confusion matrix results of the three-class classification model after feature extraction**

**(a: 1D-CNN, b: ResBiLSTM, c: 1DCNN-ResBiLSTM-Attention).**

The specific process of pixel extraction:

(I) Use the open function to open the “json” file and encode the category label information from the “json” file. Unassigned regions are labeled as “Background” with an encoding of 0, regions labeled as “Red ginseng” are assigned the code 1, and regions labeled as “Mycotoxin” are assigned the code 2. Generate a mask image of the same size as the “json” file with the above label position encodings and save this information as a numpy file.

(II) Use the “np.load” function to load the “numpy” file containing the position encoding information. Use the np.where function to find the indices of elements in the numpy array with encoding “1” and “2”, which correspond to the positions of red ginseng and mycotoxin, respectively.

(III) Map the positions obtained from the numpy array to the corresponding spectra, and extract the pixels’ information for “Red ginseng” and “Mycotoxin”.

By following the above steps, the pixels of “red ginseng” and “mycotoxin” could be successfully extracted and mapped to the corresponding hyperspectral images based on the coordinate information in the “json” file.
